# Supplementary material for: Finger somatotopy is preserved after tetraplegia but deteriorates over time
Source: eLife. 2021 Oct 19;10:e67713. doi: 10.7554/eLife.67713 (PMC8575460; doi:10.7554/eLife.67713)
Supplement: Transparent reporting form [file elife-67713-transrepform1.pdf]

eLIFE
1st Floor
24 Hills Road
Cambridge CB2 1JP, UK
P 01223 855340
W elifesciences.org
T @elife

eLife's transparent reporting form

We encourage authors to provide detailed information *within their submission* to facilitate the interpretation and replication of experiments. Authors can upload supporting documentation to indicate the use of appropriate reporting guidelines for health-related research (see EQUATOR Network), life science research (see the BioSharing Information Resource), or the ARRIVE guidelines for reporting work involving animal research. Where applicable, authors should refer to any relevant reporting standards documents in this form.

If you have any questions, please consult our Journal Policies and/or contact us: editorial@elifesciences.org.

Sample-size estimation

- You should state whether an appropriate sample size was computed when the study was being designed
- You should state the statistical method of sample size computation and any required assumptions
- If no explicit power analysis was used, you should describe how you decided what sample (replicate) size (number) to use

Please outline where this information can be found within the submission (e.g., sections or figure legends), or explain why this information doesn't apply to your submission:
submission:

No explicit power analysis was used. The sample size was limited to the availability of our specialized patient population. Due to the availability of the specialized patient population tested for this study (tetraplegic spinal cord injury patients) and the difficulty in testing these patients both in terms of MRI safety (as they mostly have implants in the cervical spinal cord) and mobility issues, we were only able to recruit 14 patients to participate in this study. This sample size is in line with previous group fMRI reports using similar methods to report on hand representation in healthy controls (n = 6; Ejaz et al., 2015, Nature Neuroscience) and congenital one-handers (n = 13; Wesselink et al., 2019, eLife).

Replicates

- You should report how often each experiment was performed
- You should include a definition of biological versus technical replication
- The data obtained should be provided and sufficient information should be provided to indicate the number of independent biological and/or technical replicates
- If you encountered any outliers, you should describe how these were handled
- Criteria for exclusion/inclusion of data should be clearly stated
- High-throughput sequence data should be uploaded before submission, with a private link for reviewers provided (these are available from both GEO and ArrayExpress)

Please outline where this information can be found within the submission (e.g., sections or figure legends), or explain why this information doesn't apply to your submission:

In the travelling wave experiment, each forward and backward run was repeated twice. The travelling wave finger maps shown for each hand and task therefore represent averages over 2 forward and 2 backward scans. This is detailed in the Materials and Methods “MRI tasks” and “Travelling wave analysis” section. We further conducted a split-half reliability analysis of the Travelling wave maps, as detailed in the Materials and Methods “Spatial correspondence of finger maps over time: Dice overlap coefficient analysis” section.

For the representational similarity analysis, we acquired data in 4 independent runs. The reported inter-finger distances are crossvalidated using these 4 runs as independent crossvalidation folds, as detailed in the Materials and Methods “Representational similarity analysis” section.

The structural MRI cross-sectional spinal cord area was calculated semi-automatically for every slice and averaged over all 10 slices to obtain a single score for cross-sectional spinal cord area. This is described in detail in the Materials and Methods, section “Structural MRI analysis”, subsection “Cervical cross-sectional spinal cord area analysis”.

The structural MRI midsagittal tissue bridges analysis was conducted once per subject and care was taken to reduce potential biases. The manual segmentation for the structural midsagittal tissue bridges analysis was conducted by an experimenter who was blinded to patient identity. Jim 7.0 software (Xinapse Systems, Aldwincle, UK) was used for this manual lesion segmentation at the lesion level, for which high intra- and interobserver reliability has previously been reported (Huber et al., 2017; Pfyffer et al., 2019). This is described in detail in the Materials and Methods, section “Structural MRI analysis”, subsection “Midsaggital tissue bridges analysis”.

Inclusion and exclusion criteria are reported in the Materials and Methods “Participants” section.

Statistical reporting

- Statistical analysis methods should be described and justified
- Raw data should be presented in figures whenever informative to do so (typically when N per group is less than 10)
- For each experiment, you should identify the statistical tests used, exact values of N, definitions of center, methods of multiple test correction, and dispersion and precision measures (e.g., mean, median, SD, SEM, confidence intervals; and, for the major substantive results, a measure of effect size (e.g., Pearson's r, Cohen's d)
- Report exact p-values wherever possible alongside the summary statistics and 95% confidence intervals. These should be reported for all key questions and not only when the p-value is less than 0.05.

Please outline where this information can be found within the submission (e.g., sections or figure legends), or explain why this information doesn't apply to your submission:

Information regarding statistical tests used, values of N, methods of multiple test correction etc. can be found in the relevant analysis sub-analysis header in the Materials and Methods section. Statistical analysis methods are described in the Materials and Methods, "Statistical analysis" section. Both frequentist and Bayesian statistical analysis was performed for the main statistical analyses.

(For large datasets, or papers with a very large number of statistical tests, you may upload a single table file with tests, Ns, etc., with reference to sections in the manuscript.)

Group allocation

- Indicate how samples were allocated into experimental groups (in the case of clinical studies, please specify allocation to treatment method); if randomization was used, please also state if restricted randomization was applied
- Indicate if masking was used during group allocation, data collection and/or data analysis

Please outline where this information can be found within the submission (e.g., sections or figure legends), or explain why this information doesn't apply to your submission:

Group allocation is described in the Materials and Methods, "Participants" section.

The structural MRI analysis required manual intervention. To reduce potential biases, the experimenter performing this analysis was blinded to patient identity.

Additional data files ("source data")

- We encourage you to upload relevant additional data files, such as numerical data that are represented as a graph in a figure, or as a summary table
- Where provided, these should be in the most useful format, and they can be uploaded as "Source data" files linked to a main figure or table
- Include model definition files including the full list of parameters used
- Include code used for data analysis (e.g., R, MatLab)
- Avoid stating that data files are "available upon request"

Please indicate the figures or tables for which source data files have been provided:

No additional files have been uploaded at this stage. We have provided results of all individual participants in the study in each figure and detailed information regarding patients' demographics and clinical scores is provided in tables, thereby making all relevant data available for the reader. Further data will be made available online prior to publication.
